# Cation-Ligand Complexation Mediates the Temporal Evolution of Colloidal Fluoride Nanocrystals through Transient Aggregation

**DOI:** 10.1021/acs.nanolett.1c03131

**Published:** 2021-11-23

**Authors:** Reut Mashiach, Haim Weissman, Liat Avram, Lothar Houben, Yael Diskin-Posner, Vaishali Arunachalam, Michal Leskes, Boris Rybtchinski, Amnon Bar-Shir

**Affiliations:** † Department of Molecular Chemistry and Material Science, Weizmann Institute of Science, Rehovot 7610001, Israel; ‡ Department of Chemical Research Support, Weizmann Institute of Science, Rehovot 7610001, Israel

**Keywords:** Nanofluorides, in situ, colloids, ligands, nanocrystals, aggregation

## Abstract

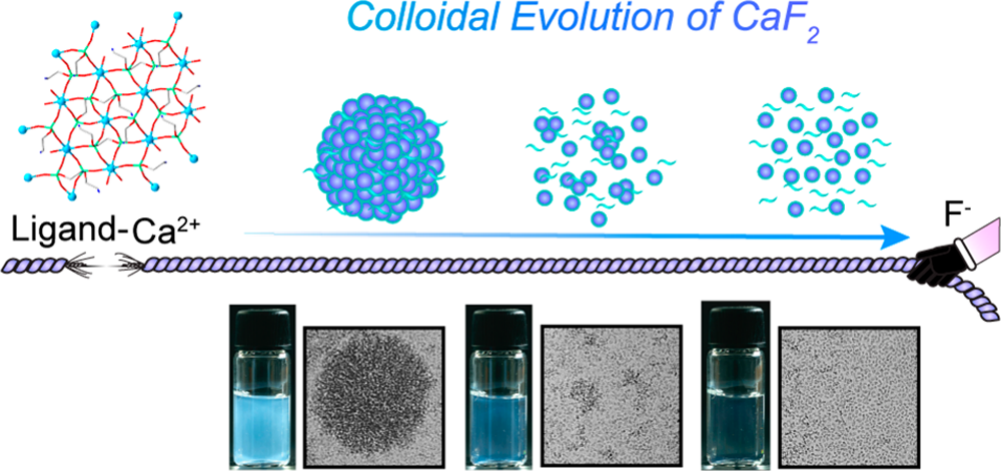

Colloidal inorganic nanofluorides have aroused great interest for various applications with their development greatly accelerated thanks to advanced synthetic approaches. Nevertheless, understanding their colloidal evolution and the factors that affect their dispersion could improve the ability to rationally design them. Here, using a multimodal *in situ* approach that combines DLS, NMR, and cryogenic-TEM, we elucidate the formation dynamics of nanofluorides in water through a transient aggregative phase. Specifically, we demonstrate that ligand-cation interactions mediate a transient aggregation of as-formed CaF_2_ nanocrystals (NCs) which governs the kinetics of the colloids’ evolution. These observations shed light on key stages through which CaF_2_ NCs are dispersed in water, highlighting fundamental aspects of nanofluorides formation mechanisms. Our findings emphasize the roles of ligands in NCs’ synthesis beyond their function as surfactants, including their ability to mediate colloidal evolution by complexing cationic precursors, and should be considered in the design of other types of NCs.

Because of their unique physical, chemical, and electronic properties,^1^ colloidal inorganic fluoride nanocrystals (NCs), namely, nanofluorides, have found applications over the last two decades as functional materials^2^ in a variety of fields, including photonics,^3^ bioimaging,^4^ biomedicine,^5^ sensing,^6^ catalysis,^7^ and more. A case in point are colloidal CaF_2_, which were developed for upconversion fluorescence,^8^ time-resolved luminescent,^9^ antifungal coating,^10^*in vivo* magnetic resonance imaging (MRI),^11−14^ prodrug activation,^15^ photodynamic therapy,^16^ remineralization of dental caries,^17^ and *in situ* NMR studies.^18^ In all these studies, the colloidal characteristics of the synthetic CaF_2_ NCs are of paramount importance to their desired properties and function. Therefore, understanding their colloidal evolution pathways in solution and identifying the key parameters that govern their dispersion dynamics are crucial for their further development and controllable design.

As is custom in the treatment of many other types of inorganic NCs, so with CaF_2_, organic ligands serve as passivating agents, stabilizing small colloids and preventing their agglomeration in solution, to allow their desired functionality.^12,19^ In addition to that role, ligands are also crucial mediators of CaF_2_ NC formation pathways, governing their growth mechanism and crystallographic features.^12,14,20,21^ In this regard, phosphate-containing ligands were found to uniquely interact with the metallic precursors in the synthetic solution via the Ca^2+^-phosphate complexes,^22,23^ as well as with the surface of the formed NC as a capping agent in several types of Ca^2+^-based NCs, even beyond CaF_2_ (including, for example, CaCO_3_^24−27^). This dual mode of interaction raises the question of phosphate-containing ligands’ role in mediating the evolution of colloidal CaF_2_ NCs in solution and, thus, can serve to control colloidal properties in the future toward a desired functionality.

Here, we demonstrate the effect of ligand–monomer interactions prior to the initiation of NC formation on the dynamic evolution of water-dispersed colloidal CaF_2_ NCs. Using a multimodal approach that combines X-ray crystallography, NMR experiments (diffusion, relaxation, and *in situ*), dynamic light scattering (DLS), and cryogenic-TEM (cryo-TEM), we show the stages through which colloidal CaF_2_ NCs evolve from a state of massive but transient aggregation to one of monodispersed, small-sized colloids in water. These observations highlight the early stages of colloidal nanofluoride formation and emphasize the role of presynthesis conditions in the evolution dynamics of colloids in water.

Upon the addition of Ca^2+^ to an aqueous solution containing the 2-ammoium ethyl phosphate (AEP) ligand (Figure 1a), a clear indication of the presence of large assemblies could be gleaned from diffusion- and relaxation- ^31^P NMR measurements. Diffusion NMR experiments revealed a notable slow diffusivity of the AEP ligand in the presence of Ca^2+^ (*D*_AEP_ = 5.34 ± 0.06 × 10^–10^ [m^2^/sec]) compared to in the presence of F^–^ (*D*_AEP_ = 5.67 ± 0.02 × 10^–10^ [m^2^/sec], Figure 1b and Table S1). This slower mobility of the ligand could be attributed to the formation of AEP-Ca^2+^ complexes in the solution in the same manner as the one shown for large aggregates^28^ and coating ligands in colloids.^29^ Moreover, the phosphate group of AEP exhibited a significantly longer spin–lattice relaxation time (*T*_1_) in the presence of Ca^2+^ (*T*_1,AEP_ = 4.36 ± 0.21 s) than in the presence of F^–^ (*T*_1,AEP_ = 2.27 ± 0.02 s, Figure 1b and Table S1). This difference in *T*_1_ values further indicates a slower tumbling rate^30^ of the obtained ligand-cation complex in water. X-ray crystallography revealed these complexes to be well-defined, ordered AEP-Ca^2+^ crystals (Figure 1c), in agreement with previous reports of AEP forming complexes with bivalent cations including Ca^2+^.^23^

**Figure 1:**
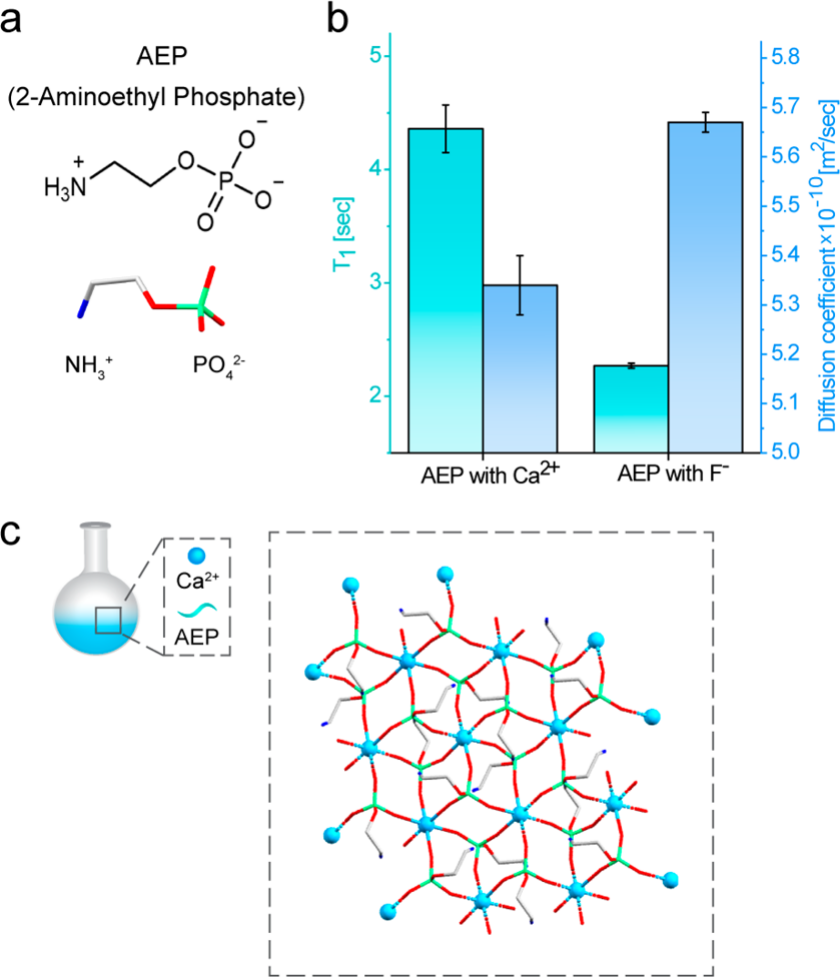
Presynthesis conditions for the synthesis of AEP-CaF_2_ NCs. (a) Molecular structure of the AEP ligand. (b) Diffusion coefficient and *T*_1_ relaxation times obtained from ^31^P NMR experiments. ^31^P-DOSY NMR experiments of the AEP ligand in the presence of Ca^2+^ and F^–^ (right axis in blue) and spin–lattice ^31^P NMR relaxation time *T*_1_ values of AEP ligands in the presence of Ca^2+^ and F^–^ (left axis in turquoise). (c) The crystal structure of the obtained Ca-AEP complex (water molecules’ representation was removed for clarification of the obtained structure).

The addition of F^–^ (as NaF solution) to the solution containing the AEP-Ca^2+^ complexes (Figure 2a) readily caused mixture turbidity, followed by a gradual diminishing of cloudiness, eventually yielding a transparent solution (Figure 2b and Movie S1). *In situ* DLS measurements of the same reaction mixture demonstrate this transition from opaqueness to transparency in a quantitative manner (Figure 2c). In these measurements, the large moieties (hydrodynamic diameter, *D*_H_ = 121 ± 46 nm, 6 min) were followed by the gradual appearance of smaller colloids (*D*_H_ = 7.9 ± 2.2 nm, 200 min). The immediate formation of the large moieties upon the addition of NaF solution (as the source of F^–^) is thus attributed to the presence of Ca-AEP complexes in the reaction solution (Figure 1c), highlighting their role in CaF_2_ colloidal evolution. This is further supported by the case where AEP-CaF_2_ NC synthesis was initiated by the addition of Ca^2+^ to a mixture of noncomplexed F^–^ and AEP (Scheme S1 and Figures S1 and S2^18^). In this case, a transparent solution of <10 nm colloids was observed over the entire course of their formation (Figure S3 and Movie S2).

**Figure 2:**
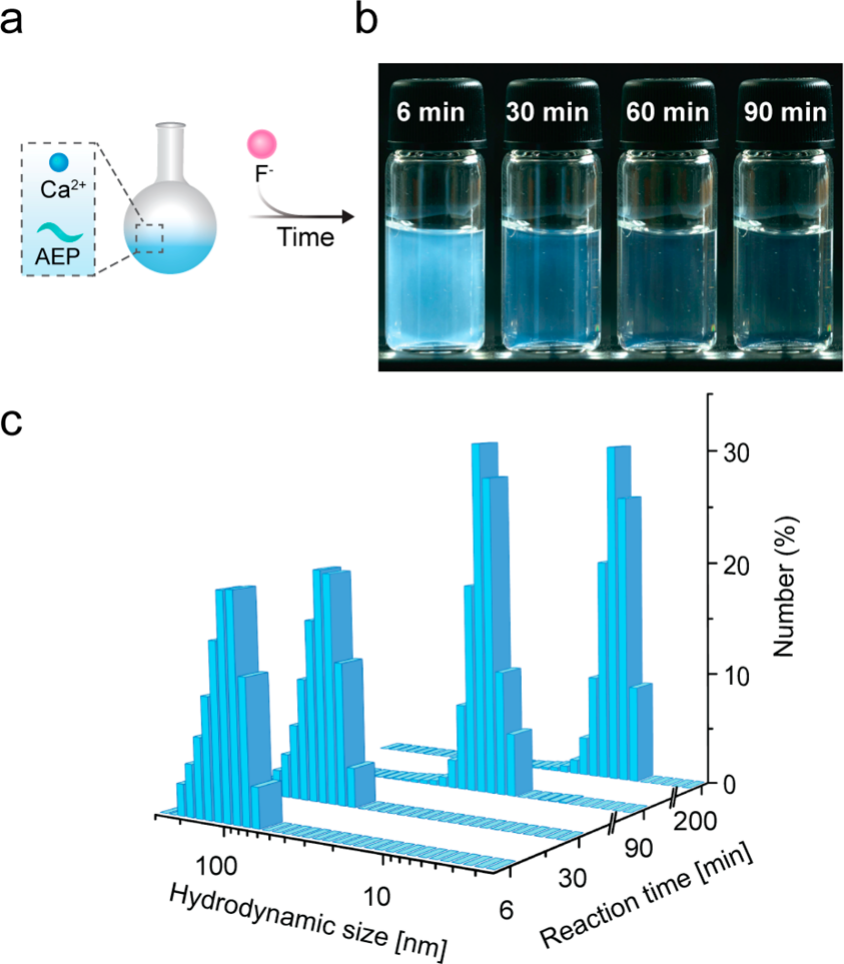
Characterization of the reaction solution of AEP-CaF_2_ NCs. (a) Schematic illustration of the synthesis of AEP-CaF_2_ NCs initiated by the addition of F^–^ (as NaF solution) to an aqueous solution of AEP-Ca^2+^. (b) An immediate cloudiness appeared upon the addition of the fluoride anions, followed by a gradual increase in transparency as the reaction progressed. (left to right) Snapshots of the reaction at 6, 30, 60, and 90 min after reaction initiation, taken from Movie S1. (c) The DLS hydrodynamic size distribution profiles of the synthesis solution at 6, 30, 90, and 200 min after reaction initiation (i.e., the addition of NaF solution).

*In situ*^31^P NMR experiments were then performed to monitor the ligand throughout the formation of the AEP-CaF_2_ NCs (Figure 3a). An immediate broadening of the ^31^P NMR peak of AEP was observed upon the addition of F^–^ suggesting their participation in the initially formed large assemblies. This noticeable line broadening was followed by a gradual narrowing of the ^31^P NMR peak in parallel to the appearance of small colloids in the studied solution (DLS, Figure 2c). Note that while DLS measurements shed light on the dynamics of the evolved colloids’ dispersity through the change in the hydrodynamic dimeter of the large contents in the reaction mixture, ^31^P NMR provides insight into the involvement of the phosphate-containing molecules in the observed process.

**Figure 3:**
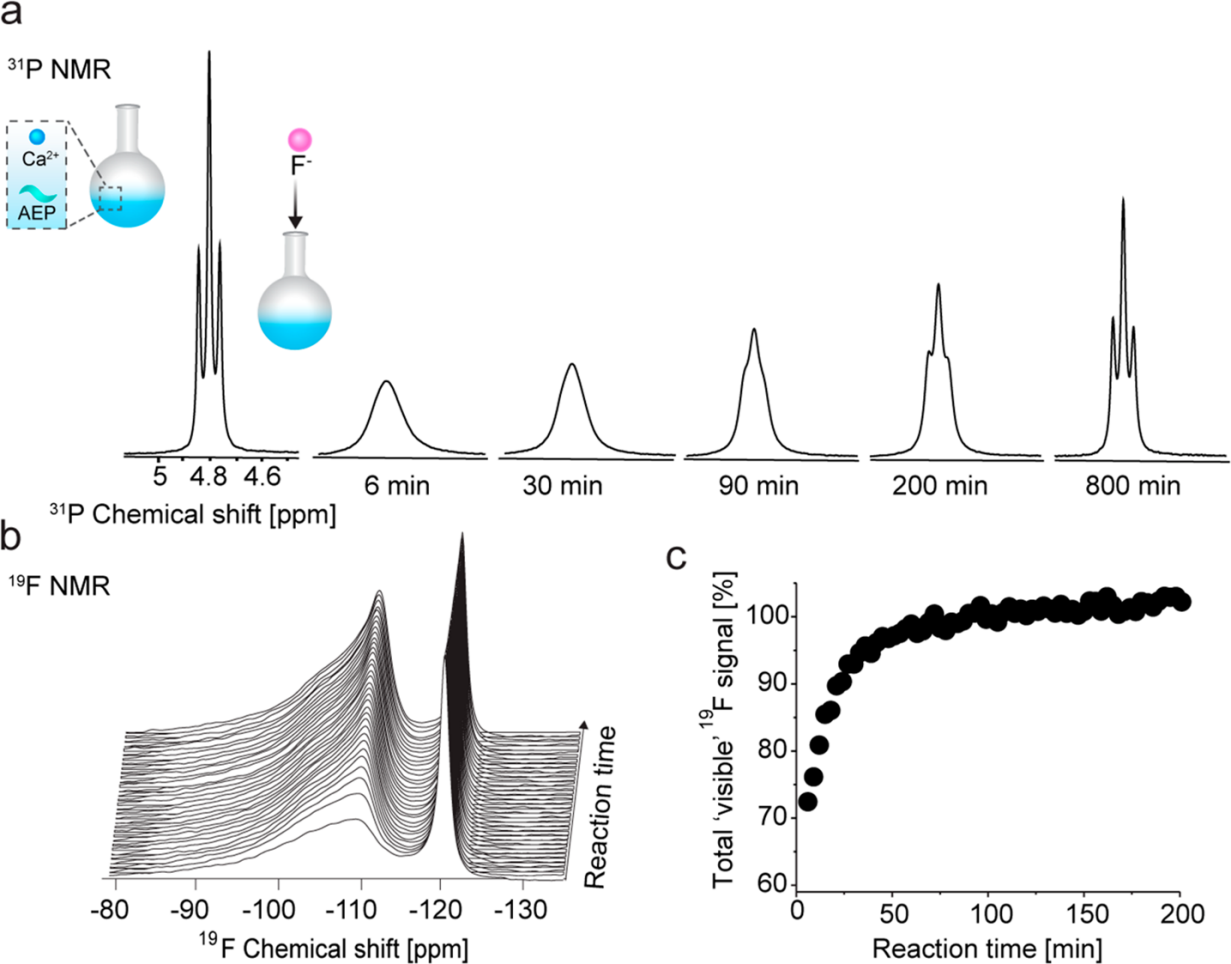
*Insitu* NMR tracking of the formation of AEP-CaF_2_ NCs in water. (a) ^31^P NMR signal of the AEP ligand before and at 6, 30, 90, 200, and 800 min after the addition of F^–^ (as NaF solution). (b) Stacked plot of the real-time ^19^F-NMR spectra of AEP-CaF_2_ synthesis, every 30 min. Each ^19^F-NMR spectrum consists of two peaks: one at −109 ppm, attributed to the fluoride of the CaF_2_ NCs, and one at −120 ppm, assigned to the free F^–^ anion in the aqueous solution. (c) Percentage of the total integrated signal of ^19^F “NMR-visible” atoms as the sum of both ^19^F resonances, −109 ppm and −120 ppm, relative to its final amount at 1000 min from the reaction initiation (synthesis end-point).

*In situ*^19^F-NMR measurements over the entire course of AEP-CaF_2_ synthesis and the subsequent quantification of the total NMR-observable ^19^F-spins indicated an initial “loss” of the ^19^F NMR signal relative to the synthesis end-point (Figure 3b,c). The undetectable ^19^F-NMR signal and its gradual recovery throughout the first 100 min of the reaction (Figure 3c) can be attributed to the restricted mobility of the initial fluoride-containing large assemblies and their continuous breakdown into smaller colloids that tumble freely in solution (Figure 2c). Moreover, setting a recovery time (TR) in the ^19^F-NMR experiment based on the *T*_1_ relaxation times of the obtained colloidal AEP-CaF_2_ NCs might not have been ideal for the *T*_1_ of some ^19^F-spins in the large assemblies at the early stages of the reaction and, thus, might have led to their filtering from the total observed ^19^F-spins.

**Figure 4:**
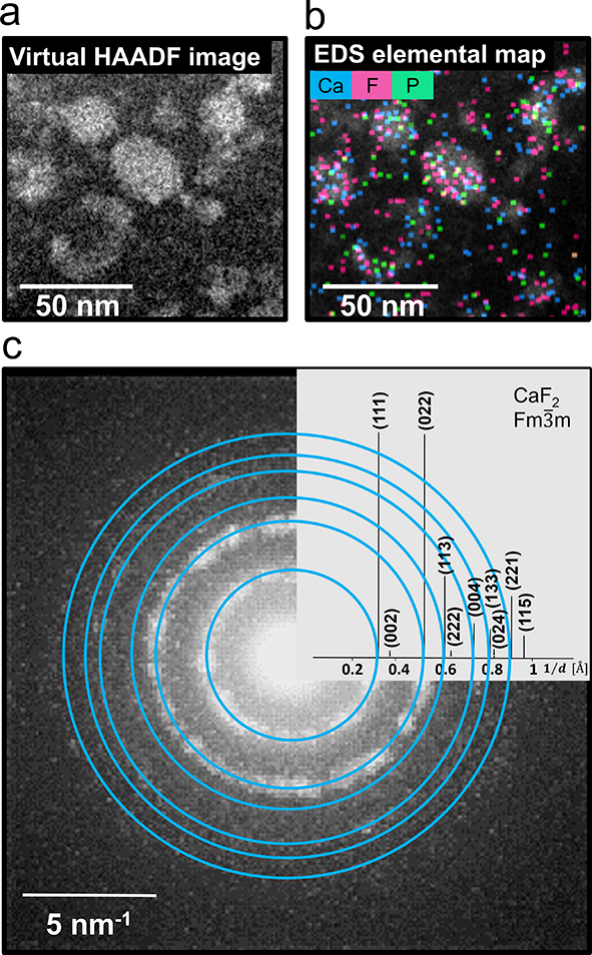
Cryo-4D STEM of the early stage of AEP-CaF_2_ NC aggregates in water (sample from the reaction solution 6 min after the addition of F^**–**^). (a) Virtual high-angle annular dark-field image (HAADF) and (b) Superimposed cryo-EDS elemental map of the large clusters of AEP-CaF_2_ elements. Calcium (blue), fluorine (pink), and phosphate (from the AEP, green) are located within the initially formed clusters. (c) Sum of the 4D STEM diffraction patterns of CaF_2_ crystals detected from within these clusters (Movie S3 and Figure S4).

**Figure 5:**
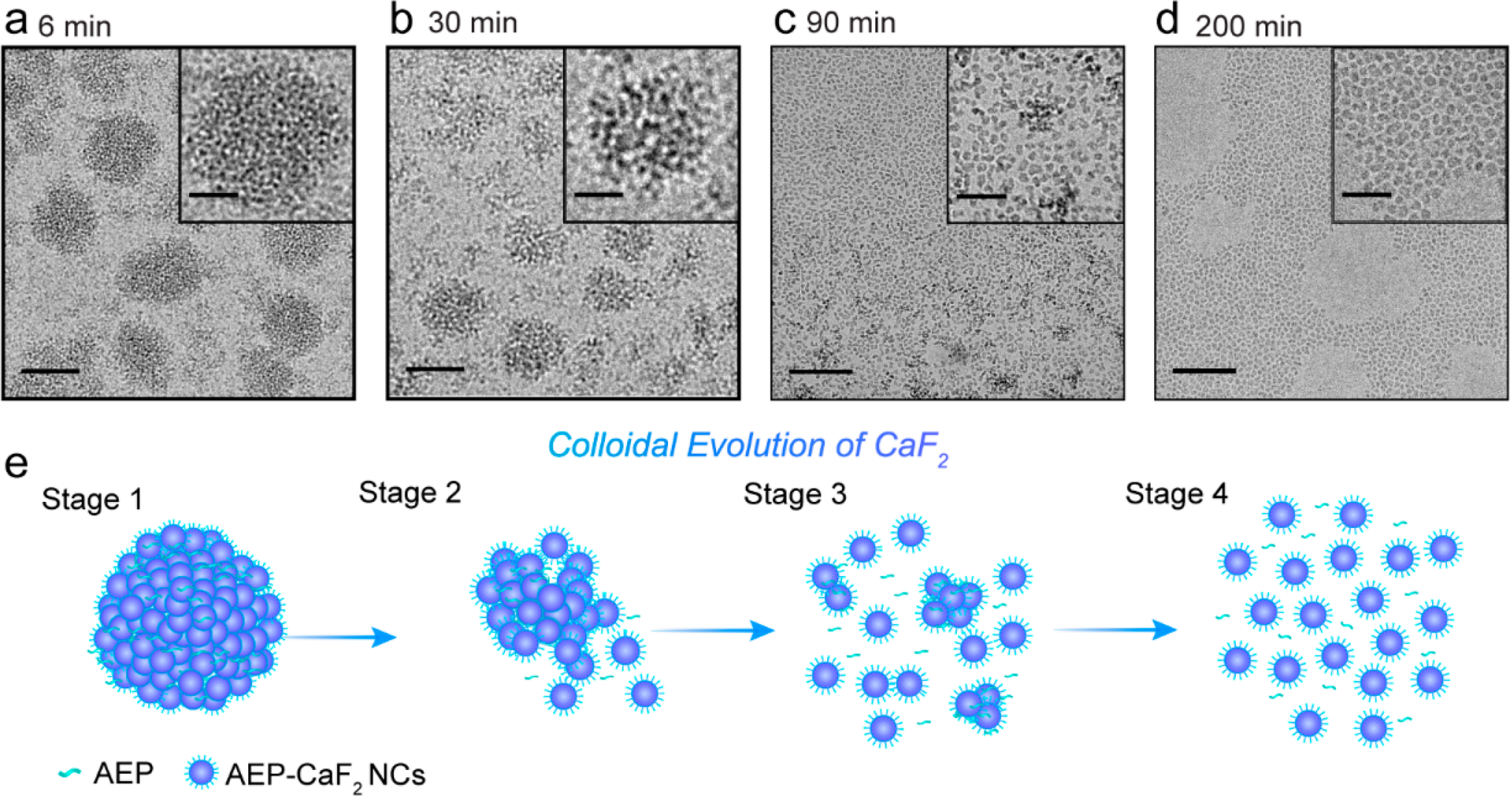
Cryo-TEM images of the colloidal evolution of AEP-CaF_2_ NCs in water, through transient aggregation. The reaction solution was sampled for cryo-TEM at four different time points: (a) 6, (b) 30, (c) 90, and (d) 200 min after reaction initiation (i.e., the addition of NaF as the source of F^–^). Scale bar, 50 nm (inset, 20 nm). (e) Schematic illustration highlighting the key stages of the colloidal evolution of AEP-CaF_2_ NCs in water.

We then employed time-resolved cryo-TEM^31^ to capture the various stages in the evolution of the AEP-CaF_2_ colloids in water in the reaction solution. Six minutes after the addition of F^–^ to the solution containing the AEP-Ca^2+^ complexes, large assemblies were depicted (Figure 4a). Energy-dispersive X-ray spectroscopy (EDS) elemental mapping confirmed the presence of Ca, F, and P elements within these structures (Figure 4b). Acquiring 4D STEM data^32^ of a region that contained the large assemblies revealed the early formation of crystals with diffraction patterns that matched CaF_2_ lattice spacings (Figure 4c, Figure S4, and Movie S3). These findings provide a direct observation that the immediately formed large assemblies contain small CaF_2_ NCs.

Representative cryo-TEM images of the same reaction mixture at different time points (i.e., 6, 30, 90, and 200 min; Figure 5a–d, respectively) revealed a dynamic process through which small colloidal AEP-CaF_2_ NCs evolve in water. The initially formed 100 nm-sized clusters (6 min, Figure 5a) disassembled to form smaller ones, as depicted 30 min from reaction initiation (Figure 5b), followed by their continuous breakdown and the appearance of small colloids (90 min, Figure 5c). At 200 min, the cryo-TEM images revealed only monodispersed small AEP-CaF_2_ NCs (Figure 5d), in good agreement with the DLS (Figure 2) and NMR (Figure 3) measurements.

Interestingly, 90 min from the reaction initiation, the cryo-TEM micrographs detected all the key stages of the colloidal evolution (Figure S5), implying that the disassembly process that occurs at this time frame could be correlated to the period where most significant ^19^F-NMR signal recovery is depicted (Figure 3c) and attributed to the release of freely tumbling colloidal NCs to the solution. Overall, these cryo-TEM data (Figure 5) show the microscopic evolution of well-dispersed colloidal CaF_2_ NCs through a transient aggregation, followed by their gradual dispersion in the aqueous solution in which they were formed. Note here, that when the same AEP-CaF_2_ NCs were formed through the addition of solution of Ca^2+^ to the mixture of noncomplexed AEP + F^–^, similar colloids were formed with no evidence of transient aggregation^18^ as the one shown above (Figures 2–5, Figure S6, and Figure S7). This finding suggests the favorable thermodynamic form of AEP-CaF_2_ can be obtained through different kinetic pathways (Scheme S1 and Figure S6).

In summary, by using a multimodal *in situ* approach (DLS, NMR, and TEM), we could create a composite view to demonstrate that colloidal AEP-CaF_2_ NCs evolve through several key stages (schematically presented in Figure 5e). Specifically, the addition of F^–^ (as NaF solution) to a solution containing Ca-AEP complexes (a presynthesis stage, Figure 1) governs the formation of transient aggregates, followed by a cascade of colloidal dispersions (Figure 5e, stages 1–4). This cascade can be explained by the dual role of F^–^, serving as a precursor to the formation of CaF_2_ NCs (stage 1) and triggering the dissociation of the Ca-AEP complex (Figure 1) due to the formation of the preferable Ca–F ionic bond. As soon as the AEP ligand becomes available (i.e., dissociates from the Ca-AEP complex), it can stabilize the formed CaF_2_ NCs as colloids (confirmed by cross-polarization (CP) ^31^P{^19^F} CP-MAS ss-NMR, Figure S8) simultaneously to the size reduction of the assemblies (stage 2). Over time, the thermodynamically stable AEP-CaF_2_ NCs are dispersed in the solution as colloids (stage 3) with no evidence of large aggregates (stage 4) left at the end of the reaction. Since the availability of the AEP-ligand to stabilize the dispersed AEP-CaF_2_ colloids is essential for the kinetic profile of the aggregation disassembly, the use of other cations (such as Sr^2+^ in the synthesis of AEP-SrF_2_ colloids, Figure S9 and Figure S10) that have a different affinity to AEP or F^–^ will result in a dissimilar kinetic profile of the disassembly process. This observation emphasizes the pivotal role phosphate-containing ligands play not just in stabilizing the formed colloids or governing their growth mechanism and crystallographic features^12,18^ but also in controlling their dynamic evolution as dispersed colloids in water. This phenomenon of ligand–monomer complexation influencing the formation of inorganic colloidal nanomaterials^22,33,34^ is of high importance to the design of nanofluorides, because their colloidal characteristics are essential for their functioning as NCs with upconversion fluorescence properties^35,36^ or as ^19^F-MR imaging tracers.^11,12^ Moreover, given the ubiquity of phosphate-Ca^2+^ complexes in nature and their important role in mediating the formation of mineral crystals such as CaCO_3_^25,27,37^ our findings could also provide additional insight into nanofabrication and biomineralization processes.

## Supporting Information Available

Experimental methods, supplementary figures and supplementary tables (PDF)
Movie showing addition of F^–^ (as NaF solution) to solution containing the AEP-Ca^2+^ complexes (AVI)
Movie showing addition of Ca^2+^ to solution containing AEP and F^–^ (AVI)
Movie showing all diffraction patterns from the ROI (MP4)

## Supplementary Material

nl1c03131_si_001.pdf

nl1c03131_si_002.avi

nl1c03131_si_003.avi

## Accession Codes

The X-ray crystallographic coordinates for the structures reported in this study have been deposited at the Cambridge Crystallographic Data Centre (CCDC), under deposition numbers 2100378–2100379. The data can be obtained free of charge from The Cambridge Crystallographic Data Centre via https://www.ccdc.cam.ac.uk/data_request/cifwww.ccdc.cam.ac.uk/data_request/cif.
