# Supplementary material for: Cation-Ligand Complexation Mediates the Temporal Evolution of Colloidal Fluoride Nanocrystals through Transient Aggregation
Source: Nano Lett. 2021 Nov 23;21(23):9916–21. doi: 10.1021/acs.nanolett.1c03131 (PMC8662719; doi:10.1021/acs.nanolett.1c03131)
Supplement: Supplementary file 1 — nl1c03131_si_001.pdf [file nl1c03131_si_001.pdf]

Supporting Information

Cation-ligand Complexation Mediates the Temporal Evolution of Colloidal Fluoride Nanocrystals through Transient Aggregation

Reut Mashiach[1], Haim Weissman[1], Liat Avram[2], Lothar Houben[2], Yael Diskin-Posner,[2] Vaishali Arunachalam[1], Michal Leskes[1], Boris Rybtchinski[1], Amnon Bar-Shir[1].*

[1]Department of Molecular Chemistry and Material Science, Weizmann Institute of Science, Rehovot 7610001, Israel. [2]Department of Chemical Research Support, Weizmann Institute of Science, Rehovot 7610001, Israel.

Supporting Methods

Sample preparation of X-ray crystallography:

AEP with Ca^{2+}: A solution containing AEP (0.17M) and $Ca(NO_3)_2$ (0.04M) in 1 ml of ddH_2O was neutralized with ammonium hydroxide to pH=7 was placed in a fume hood at 25°C for 72h to evaporate the water solvent.

AEP with F^-: A solution containing AEP (0.17M) and NaF (0.08M) in 1 ml of ddH_2O was neutralized with ammonium hydroxide to pH=7 was placed in a fume hood at 25°C for 72h to evaporate the water solvent.

Single crystal X-ray diffraction (XRD): Single crystal XRD were measured by sealed tube diffractometer Rigaku Synergy-S diffractometer dual source equipped with Dectris Pilatus3 R CdTe 300K detector and microfocus, with MoKα (λ=0.71073 Å). Data collection was performed in low temperature under LN. Data were processed with CrysAlisPRO (Rigaku). Structures were solved using SHELXT[1] and refinement performed based on F2 with SHELXL[2] and OLEX2[3] with full matrix least-squares. All non-hydrogen atoms were refined aniostropically. Hydrogens were placed at calculated positions and refined using a riding model. See crystallographic details in the Table S2. Figures were performed with CrystalMaker.[4]

Synthesis of AEP-CaF_2 NCs

Synthesis Route 1: A solution containing AEP (0.17M) and $Ca(NO_3)_2$ (0.04M) in 1 ml of ddH_2O was neutralized with ammonium hydroxide to pH=7. Next, NaF (0.08M) was injected and immediately vortexed for 3 sec. The reaction solution was either sampled at different time points for cryo-TEM measurements or was placed inside a 5 mm NMR tube for *in-situ* ^{31}P-NMR / ^{19}F-NMR experiments. (Scheme S1a)

Synthesis Route 2: A solution containing AEP (0.17M) and NaF (0.08M) in 1 ml of ddH_2O was neutralized with ammonium hydroxide to pH=7. Next, $Ca(NO_3)_2$ (0.04M) was injected and immediately vortexed for 3 sec. The reaction solution was either sampled for cryo-TEM measurements or was placed inside a 5 mm NMR tube for *in-situ* ^{31}P-NMR / ^{19}F-NMR experiments. (Scheme S1b).

Cryo-TEM

Cryo-TEM specimens were prepared by applying 6.5 µl sample (according to the two synthetic routes investigated following the injection of the second precursor) to a 200-mesh copper grid coated with holey carbon (Pacific Grid-Tech supplies). The grids were subjected to a 1 min glow discharge before sample application. Samples were blotted at 22°C and 95% relative humidity and then plunged into melting ethane using a Leica EM-GP Automatic Grid Plunger. Specimens were equilibrated at -178°C in the microscope prior to imaging. Cryo-TEM was performed on a Thermo-Fisher Scientific (TFS) Tecnai F20 twin-transmission electron microscope operating at an acceleration voltage of 200 kV. Cryogenically preserved samples were loaded into a Gatan 626 cryoholder. Low-dose TEM images were recorded with a Gatan US4000 CCD. Three samples of AEP-CaF_2 via route1 were prepared: at time points of 6, 30 and 90 min after the injection of Ca^{+2} into a solution containing both AEP and F^-. Additional Cryo-TEM measurement, 200 min after the injection of Ca^{+2} into a solution containing both AEP and F^- was performed on a TFS Talos Arctica TEM at an acceleration voltage of 200 kV. Low-dose cryo-TEM images were recorded with a Gatan OneView 4kx4k CMOS camera.

Cryo-STEM data were taken in a double-corrected TFS Themis-Z microscope at 200 kV acceleration voltage. Plunge frozen samples were mounted in a single-tilt Gatan 914 cryo-transfer holder. 4D STEM data was recorded on an EMPAD pixel array STEM detector[5] with a frame time of 1 ms. An electron probe with a semi-convergence angle of 0.3 mrad and a beam current less than 10 pA was used for 4D STEM scanning nanodiffraction. EDS hyperspectral maps were recorded at a beam current of about 30 pA on the cryo-samples. Typical recording times for EDS maps were 5 min. The EDS hyperspectral maps were processed using Velox (Thermo Fisher Scientific Microscopy Solutions, Hillsboro, USA). All elemental maps and quantitative composition data were obtained from deconvoluted and background subtracted spectra.

High resolution (HR) TEM images

Solutions of AEP-CaF_2 NCs were prepared according to both synthetic routes. After 1000 min at 25°C, the solution was centrifuged and washed with ethanol/ddH_2O. By drop cast

deposition on an ultra-thin carbon support foil the sample were dried and prepared for HR-TEM measurements. HRTEM images (Fig.S6a inset) were acquired with Talos F200X Accelerating voltage was operated at 200 kV. HRTEM images (Fig.S6b inset) were acquired with a JEOL JEM 2100 high-resolution electron microscope. Accelerating voltage was 200 kV, Gatan Digital Micrograph, a beam source of an LaB6 thermal emission, and a bottom-mount CCD camera (Gatan Ultra scanXP 2k x 2k).

NMR Experiments

Sample for ^{31}P diffusion of AEP with Ca^{2+}: A solution containing AEP (0.17M) and $Ca(NO_3)_2$ (0.04M) in 1 ml of ddH_2O was neutralized with ammonium hydroxide to pH=7 was placed inside a 5 mm NMR tube.

Sample for ^{31}P diffusion of AEP with F^- : A solution containing AEP (0.17M) and NaF (0.08M) in 1 ml of ddH_2O was neutralized with ammonium hydroxide to pH=7.

^{31}P diffusion NMR measurements were conducted on a 9.4 T (162.06566 MHz) AVANCE III NMR spectrometer (Bruker, Germany) equipped with a 50gauss/cm Z gradient system. For both samples a stimulated echo sequence with bipolar pulsed pair gradients (BPSTE) was used at 25°C. The diffusion experiments were performed with smoothed square (SMSQ.10.100) gradients with a total duration (δ) of 6ms and a recovery time of 1ms resulting in a TE of 16ms. The gradients were incremented from 2% to 98% in 10 linear steps and the diffusion time was 80ms. The data was acquired with 16 scans and a relaxation delay of 12s resulting in a 35 minutes experiment time. Each experiment was repeated 3 times and the diffusion coefficients are the average ± standard deviation of these 3 experiments. To eliminate differences in temperature/viscosity/calibration of gradients the diffusion coefficient of the water signal was measured (^{1}H diffusion $\delta = 2$ms diffusion time was 60ms) and compared to the literature value of water diffusion coefficient. The differences between the measured value and the literature value were used as a factor, which was implemented in the calculation of the reported ^{31}P diffusion coefficient for each sample. Calibrated values are reported in Table S1.

^{31}P *in-situ* NMR measurements were conducted on a 9.4 T AVANCE III NMR spectrometer (Bruker, Germany) at 25°C (162.06566 MHz). Each NMR spectrum was measured with a spectral width of 100.17 ppm and a frequency offset of 561Hz. The data was collected with 32K points zero filled to 128K. The acquisition time was 1.01 s and a relaxation delay of 15 s. Each experiment was measured with 12 scans resulting in a 3.25 minutes experiment time.

^{31}P T_1 NMR experiments were measured on a 9.4 T AVANCE III NMR spectrometer (Bruker, Germany) at 25°C (162.06566 MHz) by using the inversion recovery pulse sequence. The data was collected with 8 different delays ranging from 10μs to 25s. A spectral width of 100.17ppm was measured with 32K pointsand an acquisition time of 1.01s. Each experiment was measured with 8 scans and a relaxation delay of 24s, resulting in an experiment time of 33 minutes.

^{19}F *In-situ* NMR experiments were conducted on a 9.4 T NMR (376.7077 MHz) AVANCE III spectrometer (Bruker, Germany) at 25°C. To increase signal to noise a 30 degree RF pulse was used with a relaxation delay of 400ms, following the Ernst angle. The parameters were determined according to the equation $\cos(\alpha)=e^{-TR/T_1}$ with TR and T_1 set accordingly to AEP-CaF_2 NCs. A spectral width of 70ppm was collected with 16K points, zero filled to 64K. The frequency offset was -40,727Hz and the acquisition time was 320ms. Each spectrum was measured with 240 scans resulting in a 3 minutes experiment time. Quantifying the total NMR-observable ^{19}F-spins throughout the course of AEP-CaF_2 synthesis, the fluorides of the NCs (as CaF_2, resonate at -109 ppm) or fluoride anions in the solution (F^-, resonate at -120 ppm) were integrated and calibrated to the final amount at the synthesis end-point at 1000 min.

ss-NMR

Solutions of AEP-CaF_2 NCs were prepared according to both synthetic routes. After 1000 min at 25°C, the solution was centrifuged and washed with ethanol/ddH_2O. The final product was dried under nitrogen for 20 minutes and was used for solid state NMR (ss-NMR) experiments, which were performed on a 9.4 T (400 MHz) AvanceIII spectrometer

using a Bruker 1.3 mm double-resonance probe at magic angle spinning (MAS) rate of 60 kHz. Spectra were recorded within 2 scans using a single pulse excitation of 1.9µs (133 kHz nutation frequency) with a relaxation delay of 30 s between each scan. The relaxation delay was longer than 5 times the longitudinal relaxation time measured by saturation recovery. The chemical shift was references with respect to LiF standard set at - ppm (^{19}F) and ammonium dihydrogen phosphate at 0.81ppm (^{31}P). CP was performed at the double quantum condition with ramped nutation frequency of 37kHz on ^{19}F and constant nutation frequency of 32kHz on ^{31}P at variable contact times.

DLS

Dynamic light scattering measurements were performed with Malvern Nano-ZS, in a 12.5 mm diameter plastic cuvette where water is the solvent with a viscosity of 0.8827 cp. The diameter and size distribution of the dispersed moieties.

Supporting Figures

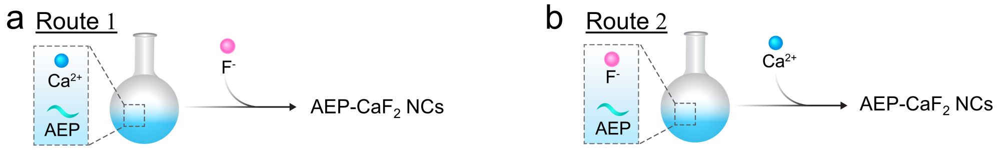

Scheme S1. **Illustration of the experimental setup of the formation of the AEP-CaF_2 NCs using two synthetic routes in water.** (a) Route 1, the main route studied in this work. Addition of F^- (as NaF solution) following the metal-ligand complexation (Ca-AEP). (b) Route 2 is the synthetic route used as a comparison where no prior complexation was detected between F^- and the AEP ligands, i.e., the addition of Ca^{2+} (as $Ca(N)_3)_2$ solution) to the solution of F^- +AEP).

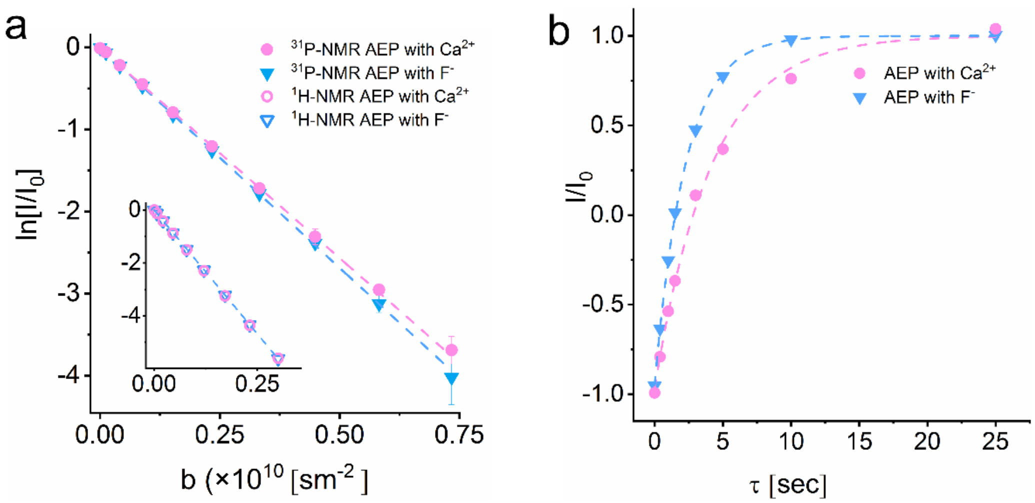

Figure S1. NMR measurements of AEP ligands in the presence of Ca^{2+} (from $Ca(NO_3)_2$) or F^- (from NaF) in solution. (a) ^{31}P-diffusion experiments of the AEP ligands in the studied samples; AEP and Ca^{2+} (pink-filled circles) and AEP and F^- (blue-filled triangles). N=3. In inset, ^{1}H-diffusion experiments of the solvent in the studied samples; AEP and Ca^{2+} (pink empty circles) and AEP and F^- (blue empty triangles). (b) ^{31}P-signals of CaF_2 NCs as a function of the inversion time obtained from ^{31}P-NMR inversion-recovery experiments performed for solutions of AEP and Ca^{2+} (pink circles) and AEP and F^- (blue triangles). Dashed lines are the fitting lines; the values are summarized in Table S1.

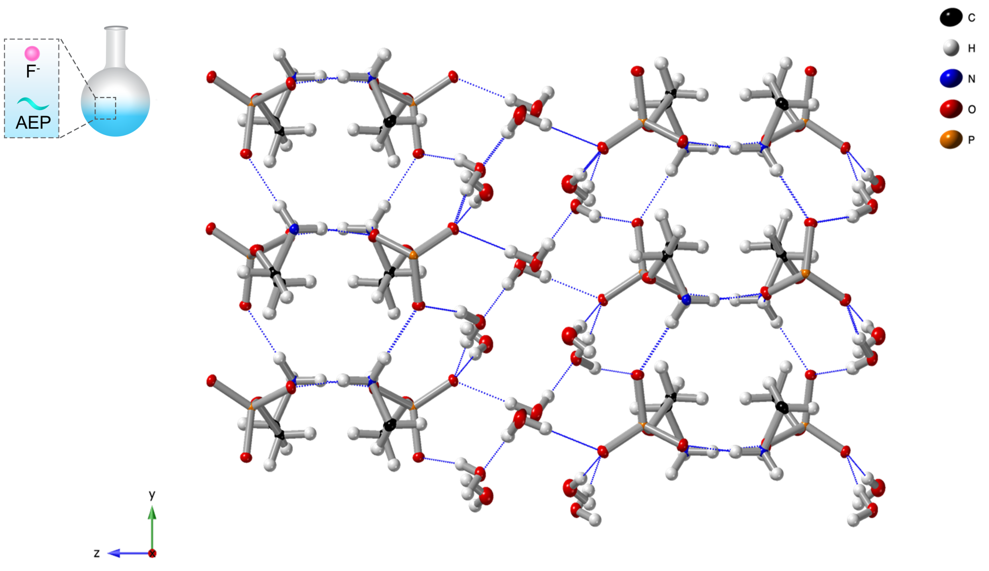

Figure S2. Crystal packing plane *bc* of structure AEP. Stacked layers of AEP are coordinated to other AEP layers and water layer. Atoms are presented as thermal ellipsoids at 50% probability level. Hydrogens are presented as spheres. Hydrogen bonds are marked in dashed blue line. The crystal of AEP was obtained from a solution containing both AEP and F^- anions.

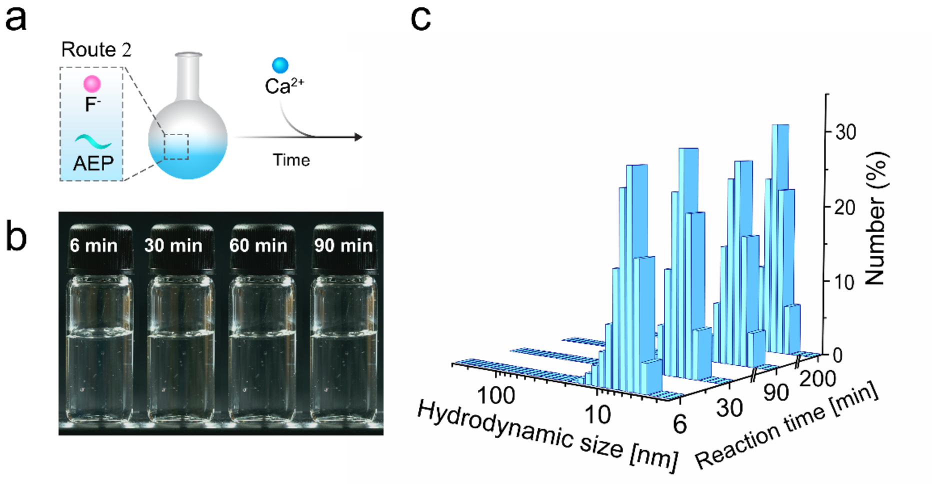

Figure S3. Characterization of the synthesis of AEP-CaF_2 upon the addition of Ca^{2+} to a non-complexed anion-AEP systems (Route 2, Scheme S1). (a) Schematic illustration for the synthesis of AEP-CaF_2 (Route 2) . (b) Clear solution throughout the reaction taken from Movie S2. Reaction times captured in: 6, 30, 60 and 90 min, left to right. (c) DLS hydrodynamic size distribution profiles of the synthesis solution at 6, 30, 90, and 200 min from the reaction initiation by the addition of Ca^{2+}.

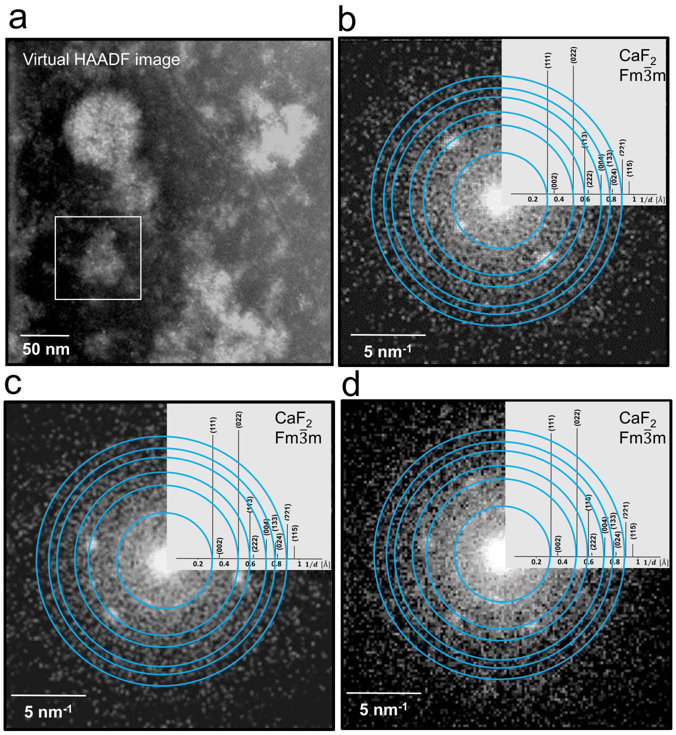

Figure S4. Early stage of AEP-CaF_2 NCs aggregates in water as detected with cryo-4D STEM (6 min from the reaction initiation upon the addition of F^-). (a) Virtual HAADF image and (b-d) individual diffraction patterns obtained within the ROI in (a) marked by a white square. The patterns reveal CaF_2 NC within the larger assemblies. Movie S3 shows all diffraction patterns from the ROI, (b-d) are a small subset similar to many more pattern showing CaF_2 single crystals.

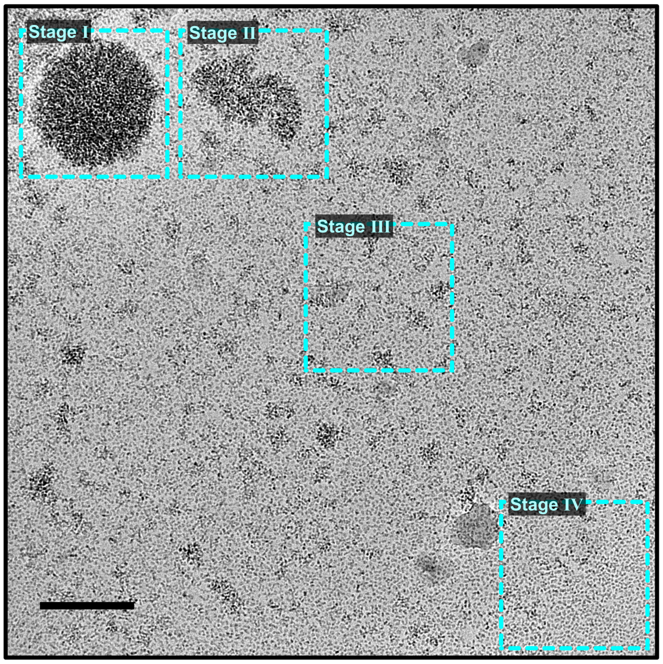

Figure S5. Cryo-TEM of the colloidal evolution of AEP-CaF_2 NCs in water as detected with cryo-TEM following metal-ligand complexation at 90 min from reaction initiation, scale bar is 100 nm.

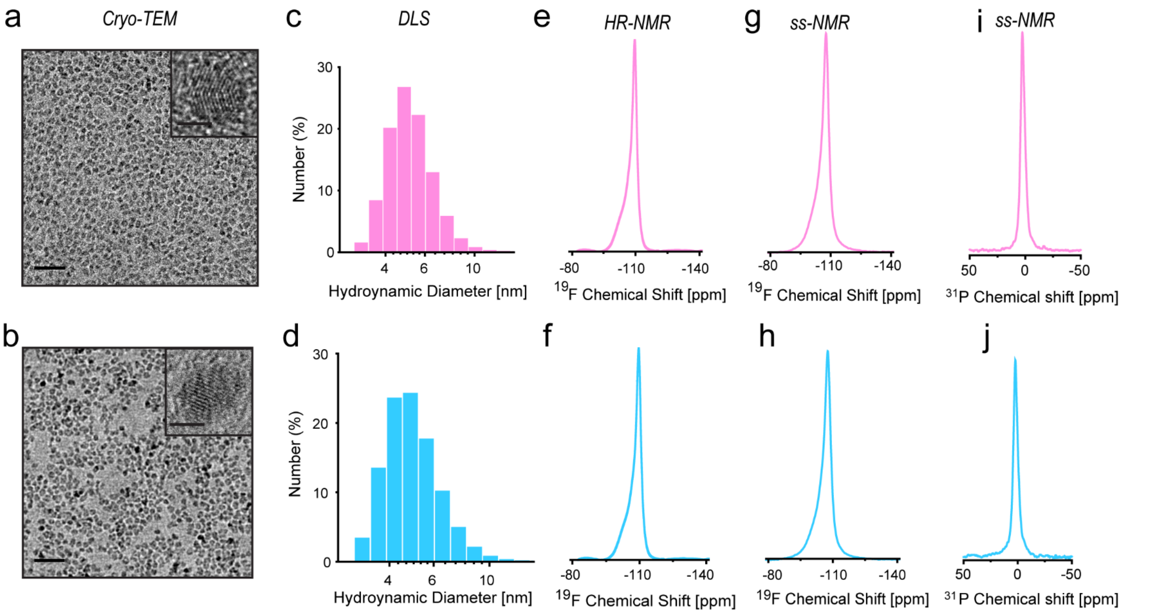

Figure S6. Characterization of colloidal AEP-CaF_2 NCs following the two synthetic routes. Top row: AEP-CaF_2 NCs synthesized through Route 1. Bottom row: AEP-CaF_2 NCs synthesized through Route 2. Cryo-TEM images (a, Route 1; b, Route 2) of colloidal NCs obtained 1000 min after the addition of the second precursor (respectively). Scale bar is 20 nm. In inset, HR-TEM images of purified colloidal AEP-CaF_2 NCs. Scale bar 2 nm. (c-d) DLS hydrodynamic size distribution profiles of the synthesis solution obtained through Route 1 (c) and Route 2 (d). (e-f) High-resolution ^{19}F-NMR (HR-NMR) of the AEP-CaF_2 NCs in water obtained from Route 1 (e) and Route 2 (f). (g-h) Solid-state ^{19}F-NMR (ss-NMR) of dried AEP-CaF_2 obtained from Route 1 (g) and Route 2 (h) with a MAS of 60 kHz. (i-j) Solid-state ^{31}P-NMR (ss-NMR) of dried AEP-CaF_2 obtained from Route 1 (i) and Route 2 (j) with a MAS of 60 kHz.

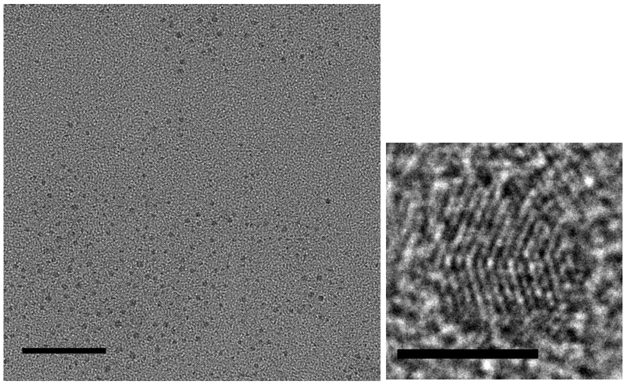

Figure S7. HR-TEM of the final stage of the synthesis of AEP-CaF_2 NCs in water (1000 min after reaction initiation) upon Route 1. Scale bars 50 nm (left image), inset 3 nm (right image).

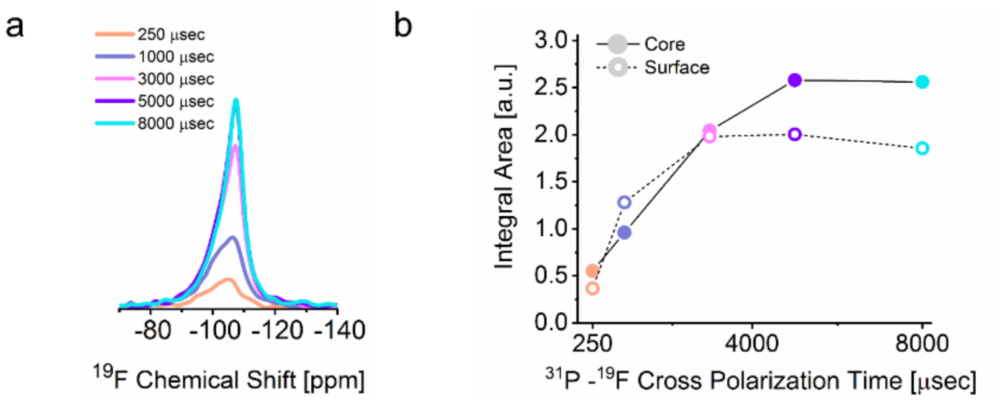

Figure S8. $^{31}P\{^{19}F\}$ CP-MAS ssNMR of purified AEP-CaF_2 NCs. (a) ^{19}F-ssNMR spectra of AEP-CaF_2 NCs obtained with cross-polarization (CP) from ^{31}P using different CP times. (b) The CP buildup of two deconvolved peaks in the ^{19}F-NMR spectrum: (*i*) at -105 ppm, marked by open circles (assigned to the ^{19}F-spins at the surface of the NC) and (*ii*) at -109 ppm, marked by filled circles (assigned to the ^{19}F-spins in the core of the NC). The lines connecting the experimental points were added for visualization only. The stronger correlation is obtained for the phosphate head group of the ligand with the fluoride content at the surface of the NCs (resonate at -105 ppm), compare to the correlation obtained with the fluorides at the core of the NCs (resonate at -109 ppm in the ^{19}F-NMR spectrum).

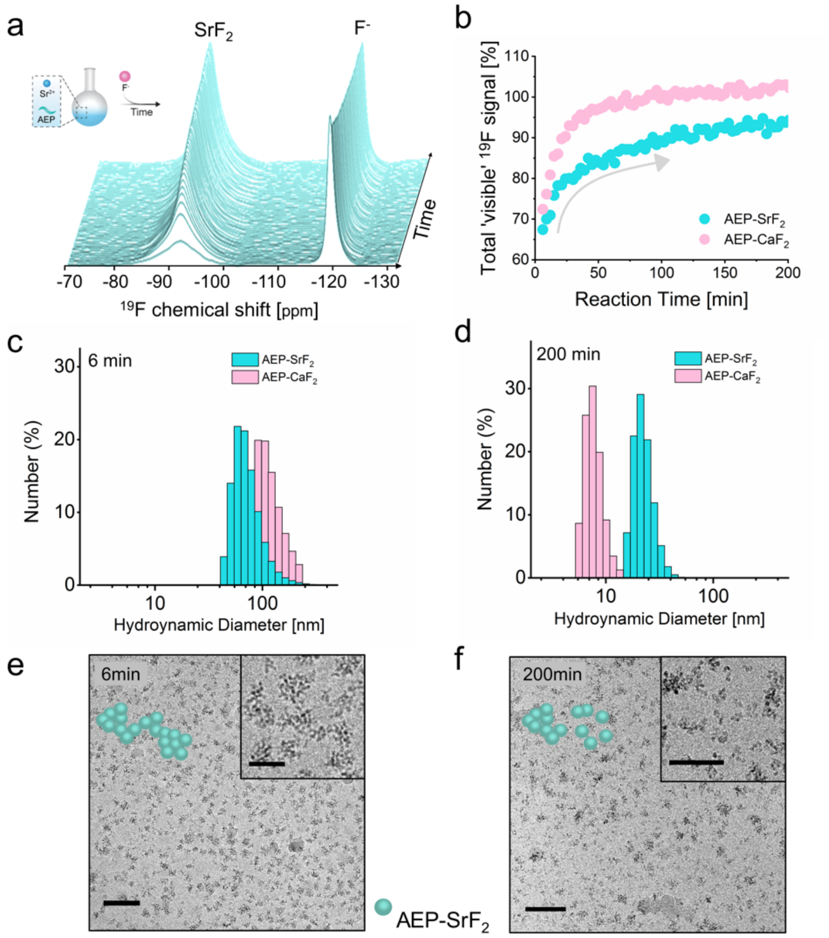

Figure S9. Synthesis progression of AEP-SrF_2 NCs in water as detected with ^{19}F-NMR, DLS and cryo-TEM via Route 1 (shown as inset in a). **(a)** Stacked plot of the real-time ^{19}F-NMR spectra of AEP-CaF_2 synthesis, every 30 min. Each ^{19}F-NMR spectrum consists of two peaks: one at -88 ppm, attributed to the fluoride of the SrF_2 NCs, and one at -120 ppm, assigned to the free F^- anion in the aqueous solution. **(b)** Percentage of the total integrated signal of ^{19}F 'NMR-visible' atoms (turquoise) as the sum of both ^{19}F resonances, -88 ppm and -120 ppm, relative to their final amount 1000 min after reaction initiation (synthesis end-point) compared to the results obtained for AEP-CaF_2 formation (pink). (c-d) DLS hydrodynamic size distribution profiles of the synthesis solution 6 and 200 min after reaction initiation by the addition of Sr^{2+} (Route 1) (turquoise columns) compared to the results obtained for AEP-CaF_2 formation (pink columns). (e-f) Samples of the AEP-SrF_2 NCs reaction mixture (Route 1) 6 min (e) and 200 min (f) after reaction initiation by the addition of F^-. Scale bars represent 100 nm (large images) and 50 nm (insets).

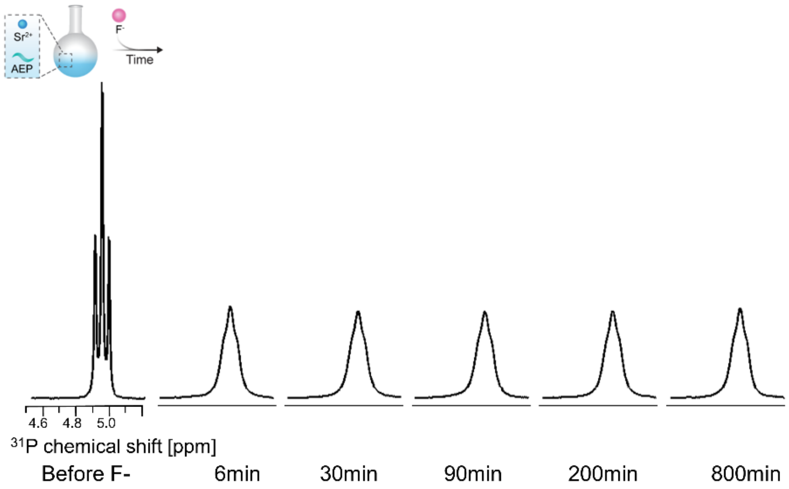

Figure S10. *In-situ* ^{31}P-NMR tracking of the formation of AEP-SrF_2 NCs in water. (a) ^{31}P-NMR signal evolution of the AEP ligand before and 6, 30, 90, 200 and 800 min after the addition of F^-.

Supporting Tables

Table S1. Diffusion and relaxation (T_1) ^{31}P-NMR. Experiments performed on solutions prior the reaction of AEP-CaF_2 initiation, i.e., the pre-synthesis conditions.

Experiment / Sample	^{31}P Diffusion coefficient $\times 10^{-10}$ [m^2/sec]	^{31}P T_1 [sec]
AEP with Ca^{2+}	5.34 ± 0.06	4.36 ± 0.21
AEP with F^-	5.67 ± 0.02	2.27 ± 0.02

Table S2. Crystallographic data of the two pre-synthesis conditions for the synthesis of AEP-CaF_2 NCs

	**AEP with Ca^{2+} (Ca-AEP)**	**AEP with F^- (AEP)**
CCDC	2100379	2100378
Empirical formula	$3C_4H_{12}CaNO_8P_2$+$2NO_3$+$2H_4N$+$1.02H_2O$	$C_2H_7NO_4P + 3H_2O$
Crystal description	Colorless plate	Colorless plate
Formula weight (g/mol)	1230.93	194.10
Temperature (K)	100(2)	100(2)
Crystal system	Trigonal	Orthorhombic
Space group	*R-3*	*Pbca*
a(Å)	10. 0202(3)	7.6343(5)
b (Å)	10.0202(3)	9.0264(6)
c (Å)	39.5343(18)	24.7574(16)
α (°)	90	90
β (°)	90	90
γ (°)	120	90
Volume ($Å^3$)	3437.6(3)	1706.04(19)
Z	3	8
Density (calculated) (Mg/m^3)	1.784	1.511
Absorption coefficient (mm^{-1})	0.687	0.323
F(000)	1930	824
Crystal size (mm^3)	0.253 x 0.102 x 0.033	0.187 x 0.103 x 0.068
Theta range for data collection (°)	1.55 to 28.27	3.14 to 28.27
Reflections collected	17080	11711
Independent reflections	1915 [R(int) = 0.0319]	2113 [R(int) = 0.0484]
Completeness (to theta = °)	100.0% (28.22)	99.9% (28.22)
Data / restraints / parameters	1915 / 22 / 137	2113 / 2 / 117
Goodness-of-fit on F^2	1.081	1.057
Final R indices [I>2sigma(I)]	R1 = 0.0316, wR2 = 0.0848	R1 = 0.0484, wR2 = 0.1259
R indices (all data)	R1 = 0.0363, wR2 = 0.0882	R1 = 0.0548, wR2 = 0.1306
Largest diff. peak and hole (e.$Å^{-3}$)	0.604 and -0.450	0.530 and -0.625

Supporting References

1. Sheldrick, G. M., SHELXT - integrated space-group and crystal-structure determination. *Acta Crystallogr A Found Adv* **2015,** *71* (Pt 1), 3-8.
2. Sheldrick, G. M., Crystal structure refinement with SHELXL. *Acta Crystallogr C Struct Chem* **2015,** *71* (Pt 1), 3-8.
3. Dolomanov, O. V.; Bourhis, L. J.; Gildea, R. J.; Howard, J. A. K.; Puschmann, H., OLEX2: a complete structure solution, refinement and analysis program. *Journal of Applied Crystallography* **2009,** *42* (2), 339-341.
4. Palmer, D. C., CrystalMaker. *CrystalMaker Software Ltd.* Begbroke, Oxfordshire, England **2014**.
5. Tate, M. W.; Purohit, P.; Chamberlain, D.; Nguyen, K. X.; Hovden, R.; Chang, C. S.; Deb, P.; Turgut, E.; Heron, J. T.; Schlom, D. G.; Ralph, D. C.; Fuchs, G. D.; Shanks, K. S.; Philipp, H. T.; Muller, D. A.; Gruner, S. M., High Dynamic Range Pixel Array Detector for Scanning Transmission Electron Microscopy. *Microscopy and Microanalysis* **2016,** *22* (1), 237-249.
